# Testing the potential of proposed DNA barcoding markers in *Nezara virudula* and *Nezara antennata* when geographic variation and closely related species were considered

**DOI:** 10.1093/jis/14.1.79

**Published:** 2014-01-01

**Authors:** Min Li, Qiang Liu, Li Xi, Yang Liu, Gengping Zhu, Yanni Zhao, Wenjun Bu

**Affiliations:** 1 Tianjin Key Laboratory of Animal and Plant Resistance, Tianjin Normal University. 300387, Tianjin, China; 2 Institute of Entomology, College of Life Sciences, Nankai University, Tianjin, 300071, China

**Keywords:** 16S rDNA, *COI*, *Cyt b*, molecular markers

## Abstract

The *COI* gene as the core of the DNA barcoding system for animals has received significant attention. The observed wide overlap between intraand interspecific sequence variability has led researchers to envisage the primary *COI* -based method. The sequences of 16S rDNA, *COI* , and *Cyt b* genes of *Nezara virudula* (L.) (Hemiptera: Pentatomidae) from 13 countries and the same sequences of *N. antennata* Scott were chosen as molecular markers to analyze the intraand interspecific relationships between the closely related species in this study. The results support that *Cyt b* gene may be a good candidate alongside *COI,* when the combined factors of geographic variation and closely related species are taken into account.

## Introduction

DNA barcoding is designed to provide rapid, accurate, and automatable species identifications by using short, standardized gene regions as internal species tags ( Hebert and Gregory 2005 ). It has become a hotspot problem in biological taxonomy and the focus of controversy. The *COI* gene as the core of the global bio-identification system for animals has suffered great disputations ( Will and Rubinoff 2004 ; DeSalle 2005 ; Hurst and Jiggins 2005 ; Meier et al. 2006 ; Memon et al . 2006 , Jansen et al. 2009 ; Sundberg et al . 2010 ; Yassin et al . 2010 ). The most important problem is the observed wide overlap between intra- and interspecific sequence variability using *COI* as the molecular marker ( Meyer and Paulay 2005 ; Meier et al . 2006 ; Memon et al . 2006 ; Alexander et al. 2009 ; Jansen et al . 2009 ). The lack of resolving power of the *COI* sequence has led researchers to envisage the primary *COI* -based method. Hebert and Gregory (2005) concluded that though DNA barcoding does not assure complete taxonomic resolution using a single gene region, in many cases when it fails the results can still be resolved fully with additional genetic or other data. Other genes, such as 16S rDNA ( Vences et al . 2004 ; Steinke et al. 2005 ; Kappner and Bieler 2006 ; Aliabadian et al . 2009 ) and *Cyt b* ( Bradley and Baker 2001 ; Pfunder et al . 2004 ; DeSalle et al . 2005 ; Hajibabaei et al . 2007 ), have been also advocated as standard or as complementary DNA barcoding markers.

When considering complementary barcoding markers, geographic variation and closely related species are factors that both need to be taken into account. Species with a wide geographic distribution often contain a great amount of genetic variability. This variability is not considered by only sampling individuals from a single site, so the distinctness of the species barcode is easily underestimated ( Moritz and Cicero 2004 ; Will and Rubinoff 2004 ; Prendini 2005 ). Conversely, sequence divergences would be overestimated if the closely related species between congeneric taxa were not included ( Meyer and Paulay 2005 ). The DNA barcode database depends on the exhaustiveness of intra-taxon sampling and closely related species selecting. This point stresses a key challenge for the DNA barcoding initiative ( Frezal and Leblois 2008 ).

We approached this issue by studying the southern green stink bug, *Nezara viridula* (L.) (Hemiptera: Pentatomidae) and the oriental green stink bug, *N. antennata* Scott, which are closely related species in morphology (the genus *Nezara* Amyot and Serville only includes three species in China: *N. viridula* , *N. antennata* , and *N. yunnana* ). *Nezara viridula* is a polymorphic and cosmopolitan pentatomid pest that causes economic damage to many crop species ( Panizzi 2000 ; Reid 2006 ; Knight and Gurr 2007 ; Martin et al. 2007 ). It is present throughout tropical and subtropical regions of Eurasia, Africa, Australia, and the Americas, located in the latitude between 45°N and 45°S, and is an active invading species ( Hoffman et al. 1987 ; Panizzi 2000 ). There are a lot of studies on the population differentiation of *N. viridula* from different countries ( Kiritani and Yukawa 1963 ; Bao-ying et al. 2000 ; Meglič et al. 2001 ; Sosa-Gómez et al. 2005 ; Kavar et al 2006 ) . Based on the current studies , the cryptic species from Botswana has been questioned ( Kavar et al. 2006 ). *Nezara antennata* is distributed mainly in the oriental region and the southeastern edge of the Palaearctic region. It is very closely related to *N. virudula* , which had been considered as the synonym for *N. viridula* ( Yang 1962 ), but studies based on interspecific copulation behavior ( Kon et al. 1993 ), pheromones ( Aldrich et al. 1993 ), and acoustical signals (songs) ( Kon et al. 1998 ) between *N. antennata* and *N. viridula* showed that they are two distinct species.

We used the sequences of 16S rDNA, *COI* , and *Cyt b* of *N. viridula* drawn from 13 countries and the same sequences of *N. antennata* to analyze the intra- and interspecific relationships between these closely related species, seeking the proper molecular marker for DNA barcoding from these three genes.

## Materials and Methods

Data for *N. viridula* from 11 different countries and regions (Slovenia, France, Greece, Italy, Madeira, Japan, Guadeloupe, Galapagos, California, Brazil, and Botswana) were downloaded from GenBank (( www.ncbi.nlm.nih.gov/genbank ). The *COI* , 16S rDNA, and *Cyt b* genes were amplified in 29 individual adult *N. viridula* from nine field collections (seven provinces in China: Guangxi, Hubei, Guangdong, Guizhou, Zhejiang, Hunan, Jiangxi; and southern and northern Iran). The same genes of *N. antennata* from individuals from two provinces (Guizhou and Zhejiang, China) were generated by PCR amplification. DNA was extracted from dissected thoracic muscles following the cetyltrime thylammonium bromide protocol (CTAB- based extraction protocol) ( Doyle and Doyle 1987 ; Cullings 1992 ). The primer sequences are shown in Table 1 . Amplification reactions were performed in a 25 µL volume. Each PCR contained 2.5 µL of 10× PCR buffer, 112.5 µM Mgcl2, 10 µM of each dNTP, 2.5 unit of Taq polymerase (Takara, ( www.takarabio.com ), 10 pM of each primer, 12.5 µL of distilled water, and 2–3 µL of DNA template. The PCR thermal regime consisted 5 min initial denaturation at 94°C, followed by 35 cycles of 45 sec denaturation at 94°C, 30 sec annealing at 60°C (16S) or 50°C ( *COI, Cyt b* ), 2 min extension at 72°C, and finalized by 10 min at 72°C. Each PCR product was subsequently purified using the gel extraction kit (Biospin, ( www.bioer.com.cn ) and sequenced on an ABI PRISM 3730 automated sequencer (by Sunbio Company, ( www.sunbio.com ). The voucher specimens were deposited in the Institute of Entomology, College of Life Sciences, Nankai University, Tianjin, China. The details of the taxa and mtDNA sequences informations are shown in Table 2 .

**Table 1.**
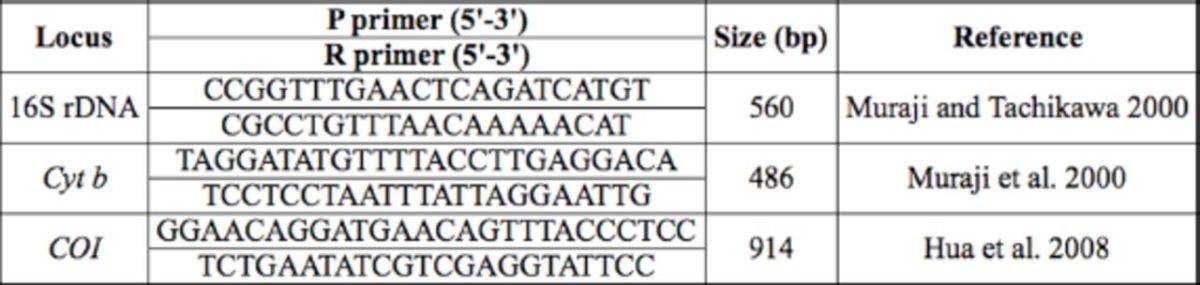
Primer used for sequence amplification.

**Table 2.**
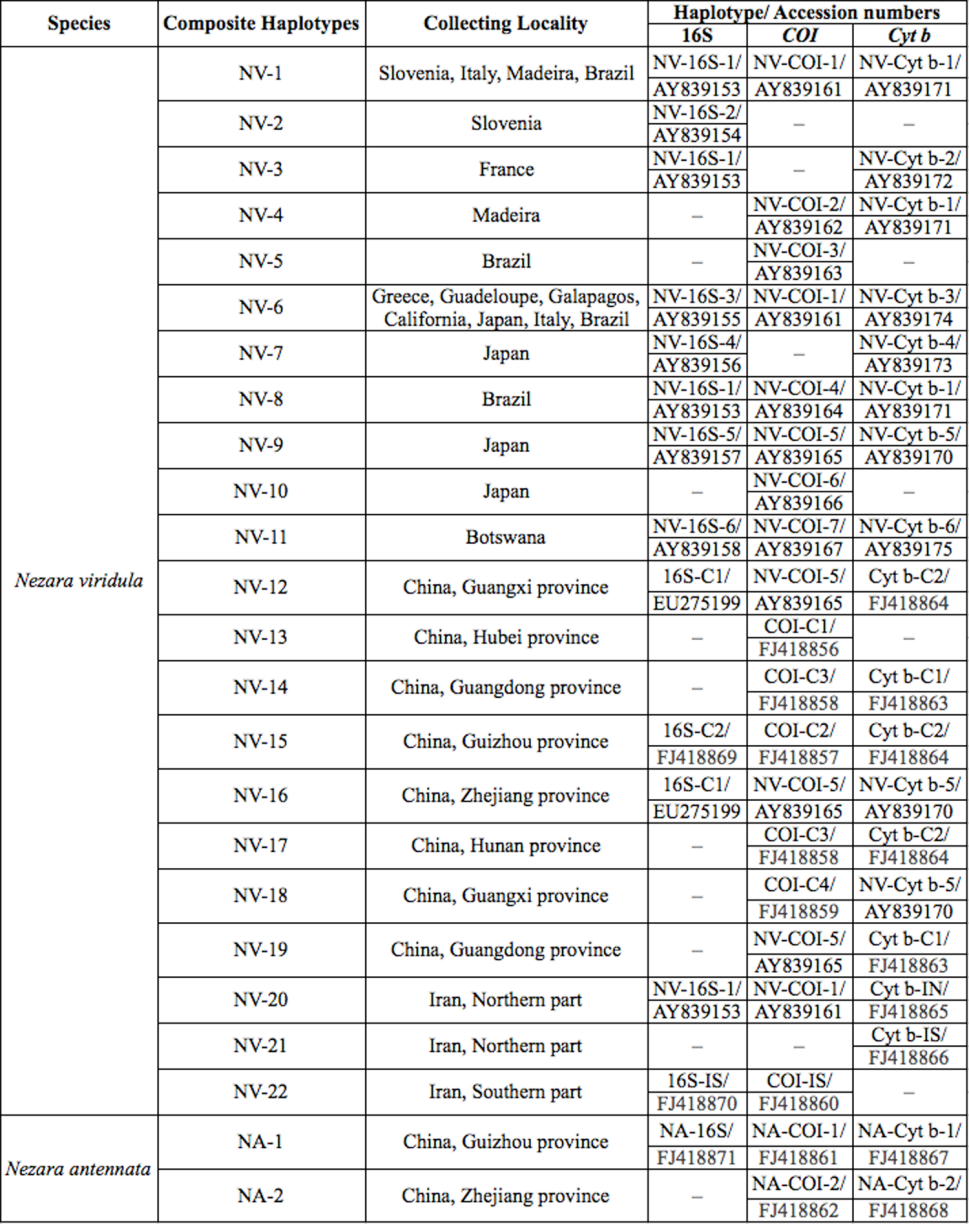
The detailed information for the *Nezara viridula* and *N. antennata* specimens used in this study. Note: The sequence accession numbers starting with EU and FJ are sequences from from this research.

Multiple sequences alignments were performed with Clustal W ( Thompson et al. 1994 ). The sequences were compared among them, and only those showing different haplotypes were included in the analysis. Mesquite version 2.74 ( Maddison and Maddison, 2010 ) was used to differentiate haplotypes of the sequences. The distinct sequences obtained in this study were submitted to GenBank, and the accession numbers are provided in Table 2 . The intraspecific genetic distances of each of three genes (16S rDNA, *COI* and *Cyt b* ) of *N. viridula* and the interspecific distance between *N. viridula* and *N* . *antennata* were calculated by Taxon DNA 1.0 ( Meier et al . 2006 ). Because the cryptic species from Botswana has been questioned by some recent studies ( Kavar et al. 2006 ), the interand intraspecific distances were re-calculated after removing the sequence from Botswana.

To construct trees, we used the neighbour joining (Saitou and Nei 1987) (Kimura 2-parameter model (K2P) for nucleotide, as recommended by Barrett and Hebert (2005)) and maximum parsimony (Swofford and Begle, 1993) methods. The methods were all performed by PAUP 4.0b for Windows (Swofford 2003). Unweighted parsimony analyses of various datasets were performed. Bootstrap values were generated in PAUP from 1000 replicates, each with 10 randomaddition heuristic searches. Sequences of two other pentatomid bugs, *Piezodorus lituratus* (F.) and *Rhaphigaster nebulosa* (Poda), were used as outgroups. As the compared sequence lengths were different from each other, it may have affected the results. We carried out separate analyses for all sequences with equal overlap. All trees were also reconstructed after removing the sequence from Botswana.

## Results

The worldwide intraspecific genetic distances of each of the three genes (16S rDNA 448 bp, *COI* 347 bp, and *Cyt b* 460 bp) of *N. viridula* from Europe, Asia, Africa, and the Americas and the interspecies distance between *N. viridula* and *N* . *antennata* are shown in Table 3 , which also contains the results after the sequence from Botswana was removed. The interspecies distances are equal or even shorter than the intraspecies distances based on each data set of three genes. The sequence differences between Botswana and other haplotypes of *N. viridula* are larger than those between *N. viridula* (without Botswana) and *N. antennata.* The overlap still existed in the results of the 16S rDNA data set even when the sequence from Botswana was removed. The ranges of the overlaps were wide (0.01– 0.03), with almost 90% of intraspecific distances falling into this interval. The interand intraspecies distances of sequences with equal length (347 bp) are shown in Table 4 with similar results of different lengths.

**Table 3.**
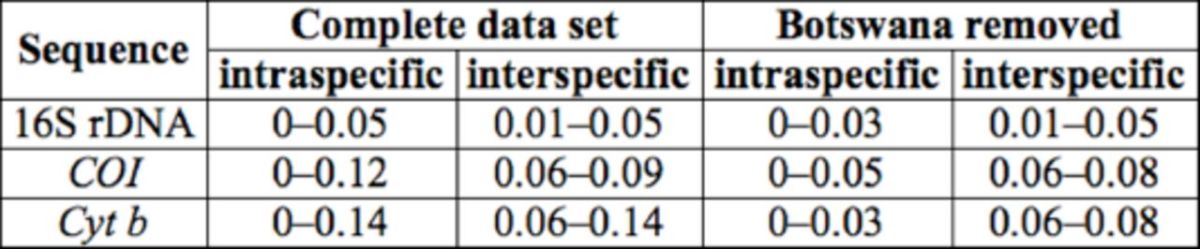
The intra- and interspecifics distance (K2P) of each gene.

**Table 4.**
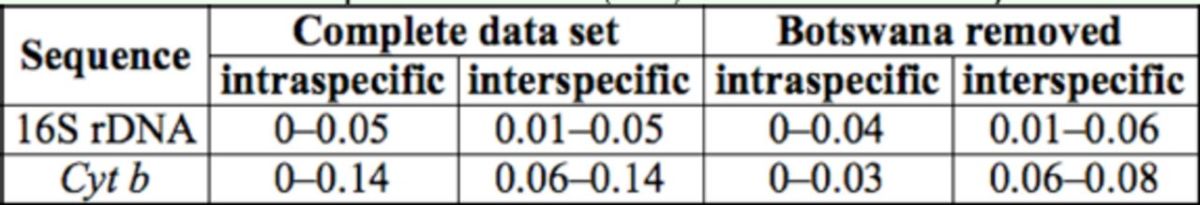
The intra- and interspecifics distance (K2P) of 16S rDNA and *Cyt b* with 347 bp.

**Figure 1.**
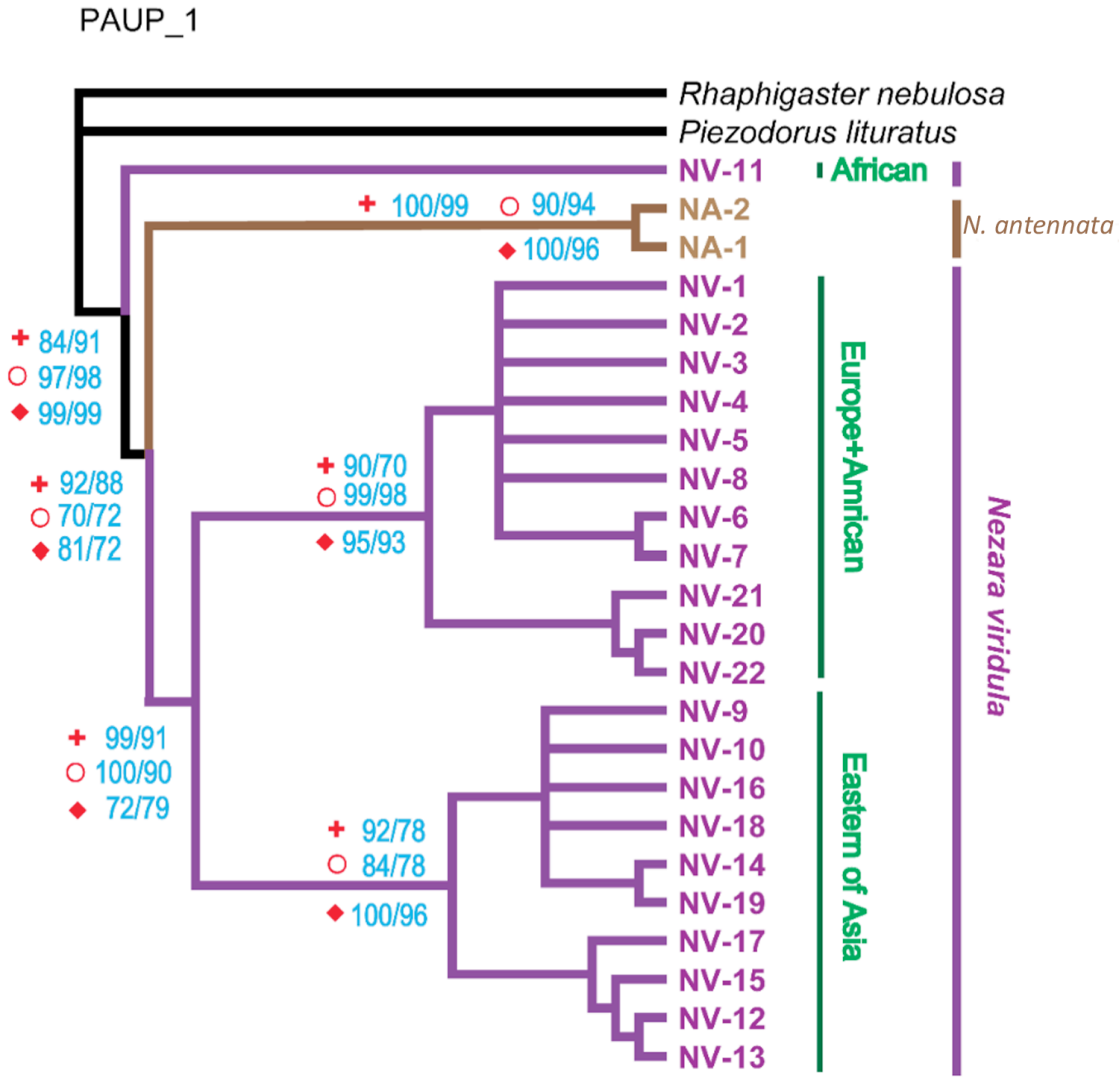
The phylogenetic tree based on three separate markers, *COI* , *Cyt b* , and 16S rDNA . The algorithms of neighbor joining and maximum parsimony (MP) methods were calculated by bootstrap resampling with 1,000 replicates. Bootstrap supports (NJ/MP) are given at the node (Symbols: plus: *COI* ; circle: *Cyt b* ; rhomb: 16S rDNA). Each data set of *COI* , *Cyt b* , or 16S rDNA (NJ and MP) produced completely resolved trees with similar topologies. High quality figures are available online.

**Figure 2.**
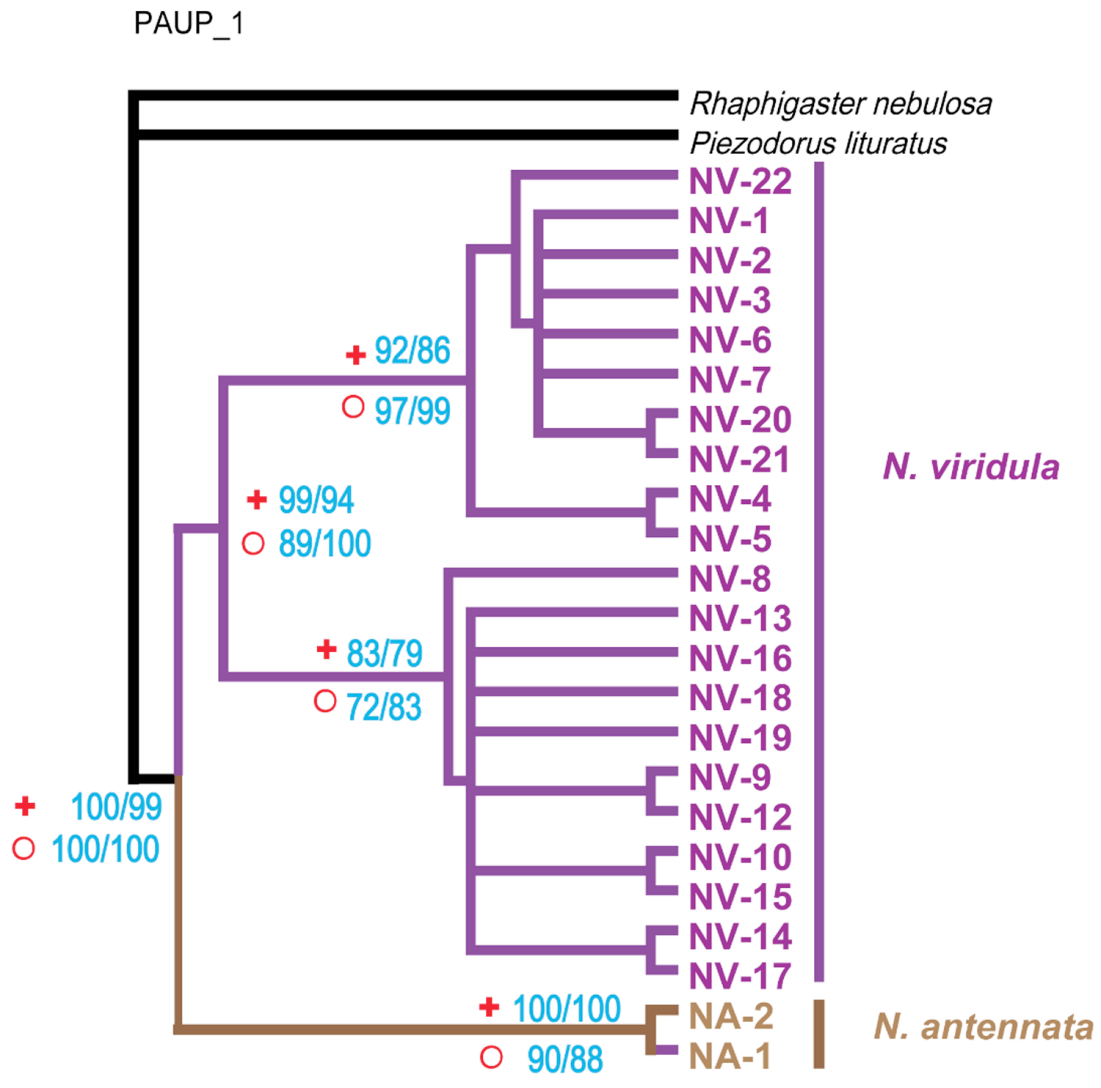
The phylogenetic tree based on separate markers *COI* and *Cyt b.* The sequence of Botswana (NV-11) was excluded. The algorithms of neighbor-joining (NJ) and maximum parsimony (MP) methods were calculated by bootstrap resampling with 1,000 replicates. Bootstrap supports (NJ/MP) are given at the node (Symbols: plus: *COI* ; circle: *Cyt b* ). Each data set of *COI* or *Cyt b* (NJ and MP) produced completely resolved trees with similar topologies. High quality figures are available online.

According to the tree reconstruction methods, the sequences of *N. viridula* and *N* . *antennata* fail to form speciesspecific clusters ( Figure 1 ). When the sequence from Botswana (Nv11) was removed, the failures still existed in the 16S rDNA data set, but *COI* and *Cyt b* are considered successfully identified, as they clustered with conspecific sequences ( Figure 2 ). The trees with a sequence of equal length (347 bp) have similar results with the different lengths data matrix.

## Discussion

Both the distanceand tree-based results suggest that the specimen from Botswana may represent a distinct species. We found that the interspecies distances were equal or even shorter than the intraspecies distances based on each data set of three genes in the worldwide populations of *N. viridula* ( Tables 3 and 4 ). The differences between sequences of *N.viridula* (without Botswana) and *N. antennata* were even smaller than those between Botswana and other haplotypes of *N. viridula.* Differences also could be found in the sexual communication system of *N. viridula,* which suggests that a cryptic species might exist ( Ryan et al. 1996 ; Jeraj and Walter 1998 ) . Further sampling in Africa will be necessary, and its reproductive isolation needs to be observed in order to ascertain its taxonomic status.

We found that the results changed when the Botswana sequence was removed ( Tables 3 and 4 ). But, the overlap and failures also existed in 16S rDNA, making it difficult to identify candidate species. The proper marker should show small sequence divergences between intraspecifics and larger distance between interspecifics, and form speciesspecific clusters. So, *16S* rDNA may not be a proper barcoding marker in this group.

In the populations of *N. viridula* (Botswana specimen (Nv-11) removed), *COI* and *Cyt b* are considered successfully identified depending on tree reconstruction techniques, as they clustered with conspecific sequences. The threshold of intraspecific variabilities of *COI* was 5%, and for *Cyt b* it was 3%. Constrained intraspecific variation is a key finding in the DNA barcode effort. Traditionally, *COI* was considered to have far less variation within species ( Hebert and Gregory 2005 ). In this study we found that the variation of *Cyt b* (0.03) within the species was smaller than *COI* (0.05). So, *Cyt b* may be a good candidate as a DNA barcoding marker along side *COI* in this group.

bHebert (2003) suggested that the thresholds of *COI* for species diagnosis are ordinarily greater than 3%. However, determining the thresholds that distinguish species in other geographical regions and taxonomic groups is important. Thresholds will particularly need to established for groups with differences in traits, such as dispersal regime or generation length, which are likely to change rates of molecular evolution or the extent of a population subdivision. What is the boundary between a population and a species? Does it exist? To solve this issue, broad-ranging intraspecific sampling should be integrated in the database, and one must consider species boundaries not as a definitive but as a revisable concept. If the geographical structure is ignored, the species delineation will be blurred and distorted.

DNA barcoding using a single gene region does not assure complete taxonomic resolution. We suggest that a number of mitochondrial and nuclear genes may be used as DNA barcoding markers to complement *COI* . *Cyt b* is a good candidate in this group.
